# Development and validity of a serious game for teaching Central Sterile Supply Department procedures

**DOI:** 10.1590/0034-7167-2025-0453

**Published:** 2026-07-24

**Authors:** Fernanda de Andrade Ribeiro Ferreira, Felipe Almeida Sales, Yure Rodrigues Silva, Maria das Neves Figueiroa, Camilla Ribeiro Lima de Farias, Felicialle Pereira da Silva, Natália Ramos Pessoa Costa, Emanuela Batista Ferreira e Pereira

**Affiliations:** I Universidade de Pernambuco. Recife, Pernambuco, Brazil

**Keywords:** Sterilization, Process Assessment, Health Care, Teaching Materials, Education, Nursing, Validation Study., Esterilización, Evaluación de Procesos, Atención de Salud, Materiales de Enseñanza, Educación en Enfermería, Estudio de Validación.

## Abstract

**Objectives:**

to construct and validate a serious game to support the teaching of the Central Sterile Supply Department physical structure, flow, and functionality in an undergraduate nursing course.

**Methods:**

a methodological study, conducted between April and August 2023, with the participation of ten expert judges and 38 nursing students. Validity was based on the serious game structure as a theoretical-methodological framework, using the Content Validity Index.

**Results:**

the “Rota CME” game simulates the physical layout of the sector for student learning and obtained an overall Content Validity Index of 98.3%. The “game structure and presentation” domain showed lower reliability (α=0.526), and was adjusted according to participants’ suggestions.

**Conclusions:**

the game demonstrated evidence of content and face validity, proving to be a viable pedagogical tool for nursing education, with the potential to enhance learning in the central sterilization unit area.

## RESUMEN

**Objetivos:**

diseñar y validar un videojuego serio para apoyar la enseñanza de la estructura física, el flujo y la funcionalidad del Departamento Central de Suministro Estéril en un curso de enfermería de pregrado.

**Métodos:**

estudio metodológico realizado entre abril y agosto de 2023, con la participación de diez jueces expertos y 38 estudiantes de enfermería. La validación se basó en la estructura del videojuego serio como marco teórico-metodológico, utilizando el Índice de Validez de Contenido.

**Resultados:**

el juego “Rota CME” simula la distribución física del sector para el aprendizaje de los estudiantes y obtuvo un Índice de Validación de Contenido general del 98,3%. El dominio “estructura y presentación del juego” mostró una fiabilidad menor (α=0,526) y se ajustó según las sugerencias de los participantes.

**Conclusiones:**

el juego demostró validez de contenido y aparente, demostrando ser una herramienta pedagógica viable para la formación en enfermería, con potencial para mejorar el aprendizaje en el área de la unidad central de esterilización.

## INTRODUCTION

The nurses’ role in the perioperative context encompasses essential activities such as organizing the environment, meeting the surgical team’s needs, and correctly processing healthcare products. These actions aim to provide surgical patients with individualized, safe care based on validated protocols, covering all stages of the care experience^(1)^. In this scenario, the Central Sterile Supply Department’s (CSSD) strategic role stands out, a unit that, although providing indirect care, has a direct impact on the safety and quality of care provided.

CSSD’s main purpose is to provide the various healthcare and diagnostic sectors with properly processed health products. To this end, strict adherence to good practices is required, involving the stages of pre-cleaning, cleaning, inspection, preparation, disinfection, sterilization, storage and distribution^(2)^. These processes are regulated by Collegiate Board Resolution (In Portuguese, *Resolução de Diretoria Colegiada* - RDC) 15/2012, which establishes criteria based on scientific evidence, fundamental to ensuring the effectiveness and safety of the practices adopted^(3)^.

A full understanding of CSSD structure, flow, and routine is indispensable for the professionals who work there, and their ability to identify nonconformities that may compromise the expected results is also essential. CSSD adequate infrastructure and physical-functional organization are key elements for the safe and efficient performance of activities. The organization of environments and the unidirectional flow of materials, from dirty to clean, contribute decisively to the prevention of adverse events, such as healthcare-associated infections^(4)^.

Due to the critical nature of the functions performed in this sector, it is essential that professional training, especially nurses, includes educational strategies that promote practical and theoretical understanding of CSSD’s specificities. However, the approach to this content in nursing education still presents limitations. A study identified that students consider it necessary to use more attractive and interactive methodologies, such as educational videos, for teaching good practices in CSSD, mainly due to the limitation of practical experiences in the sector, marked by reduced working hours and few real opportunities for immersion^(5)^.

These limitations point to gaps in training that can compromise professionals’ future performance. In this context, the use of active methodologies, such as serious games-games with educational intent-has shown promise in health education. By simulating the professional environment and activities, these games immerse students in contexts close to reality, promoting autonomy, critical thinking, and knowledge consolidation^(6,7)^.

This dialogical and participatory approach is in line with Paulo Freire’s Critical and Emancipatory Education Theory, which understands education as a process of transformation, based on critical reflection and conscious action on lived reality^(8)^. The inclusion of innovative teaching resources, such as educational games, has proven effective in promoting meaningful learning, especially in the teaching of technical and abstract concepts. These resources offer, in addition to content, observable indicators of student performance, allowing qualitative and quantitative assessment of the teaching-learning process^(9)^.

However, the effectiveness of these materials depends on a rigorous construction and validity process, conducted by experts in the field, which has been a recurring theme in research at the interface between education and health^(10)^.

## OBJECTIVES

To develop and validate a serious game about CSSD structure, flow, and functionality aimed at nursing students.

## METHODS

### Ethical aspects

The study was conducted in accordance with national and international ethical guidelines and approved by the *Universidade de Pernambuco* Research Ethics Committee, whose opinion is attached to this submission. It complies with Circular Letter 2/2021, which establishes guidelines for research procedures in a virtual environment. Additionally, an Informed Consent Form was obtained from all individuals involved in the study via a link to access the participation form, which contained the consent form on its first page, meaning that other parts of the form could only be accessed after participants’ consent. The instrument also allowed for suggestions for changes, and judges were identified by codes (P1, P2...) to ensure anonymity.

### Study design, period and location

This is a methodological study that sought to construct and validate a serious game aimed at nursing students to support teaching in CSSD. The study was conducted between April and August of 2023.

To guide the process, the COnsolidated criteria for REporting Qualitative research was used^(10)^. The stages for the construction and validity process followed the stages described by the American Educational Research Association, which considers the following validity evidence: evidence based on test content; evidence based on response processes; evidence based on internal structure; and evidence based on relationships with other variables^(11)^. Thus, evidence based on the educational technology content and on response processes was verified. The research itself was developed in three stages: serious game construction; content assessment with experts; and technology application with the target audience.

As a theoretical basis, the serious game’s framework was Paulo Freire’s Critical and Emancipatory Education Theory^(8)^, and the game’s contents were defined according to the guidelines established by the following technical documents: RDC 15/2012 from the Brazilian National Health Regulatory Agency (In Portuguese, *Agência Nacional de Vigilância Sanitária do Brasil* -ANVISA)^(12)^; Guidelines for Surgical Nursing Practices and Processing of Health Products from the Brazilian Association of Surgical Center, Anesthetic Recovery and Material and Sterilization Center Nurses^(13)^; and the ABNT NBR ISO 17665-2 standard^(14)^. These documents establish the requirements for good practices in the processing of healthcare products, and they helped guide the following topics covered in the serious game: CSSD physical structure, functionality, and flowchart.

The methodological framework for creating the educational resource in the form of a game was based on the structure of a serious game. The main objective of this type of game is to teach using various multimedia resources and materials^(7)^.

### Population and sample; inclusion and exclusion criteria

Ten judges participated in this stage, respecting Pasquali’s recommendation of at least six experts on the subject to assess the proposed content^(15)^. The judges considered the following criteria: professional category; scientific production related to the field of CSSD; and level of specialization. Initially, a systematic search of scientific literature was carried out to identify authors with relevant publications on CSSD.

Based on this analysis, experts were selected and invited to participate in the study via electronic contact, including detailed information about the research objectives and participation criteria. Furthermore, judges were selected using a non-probabilistic snowball sampling method, such that after assessment, the first judge was asked to nominate new participants, who then made further nominations until the desired sample size was reached.

To ensure that the nominated judges met the criteria adopted in this research, their curriculum vitae, available on the Brazilian National Council for Scientific and Technological Development *Lattes* Platform, was verified.

The target audience for applying the technology were students in their fifth semester of a nursing course at a public university in northeastern Brazil.

### Study protocol

In the second stage of the study, the educational technology content was assessed by expert judges for clarity and relevance. Each judge received, via email, an invitation letter to participate in the research, describing the study’s objective, method of conduct, information about the origin of the material to be assessed, and a PDF file containing high-definition images of cards and the game board along with the form. The form consisted of four sections: (1) sample characterization; (2) game’s learning objective; (3) game structure and presentation; and (4) relevance in the teaching-learning process. Assessment was carried out using questions with five response options, graded on a Likert-type scale, containing the following alternatives: agree; partially agree; neither agree nor disagree; partially disagree; and disagree.

In the third stage, the evidence of validity based on response processes was sought. To this end, the technology was applied among the target audience. The game was implemented in the institution’s skills laboratory over four days, with students divided into groups of ten to ensure greater learning.

The educational intervention followed a similar script each day. Initially, there was a theoretical-practical approach in which CSSD areas were presented through stations representing supply decontamination, preparation, sterilization, and storage. Following this, the game was implemented among students by a professor responsible for the subject and a nursing student, both of whom were involved in the educational technology production. Finally, students assessed the serious game in terms of structure and presentation, relevance to the teaching-learning process, and pertinence to CSSD’s work process. For this purpose, the data was collected in a virtual environment using a Google Forms^®^ questionnaire.

Data collection was carried out using a Likert-type opinion scale instrument with objective questions containing five response options: agree; partially agree; neither agree nor disagree; partially disagree; and disagree. The instrument also provided space for undergraduate students to suggest changes if they deemed it necessary.

### Analysis of results and statistics

The results were analyzed in a Microsoft Excel^®^ spreadsheet to calculate the overall Global Item-Content Validity Index (I-CVI) and Scale-Content Validity Index (S-CVI)^(16)^. The I-CVI was obtained by the proportion of judges who responded “agree” or “partially agree” on each item. In turn, the S-CVI was calculated by averaging the proportions of items considered valid by each judge. A minimum agreement index of 80% was adopted for validity^(17)^.

To assess the questionnaire administered to students, Cronbach’s alpha internal consistency coefficient was obtained per block as an assessment criterion for item relevance. The data were entered into a spreadsheet, and the program used to obtain the statistical calculations was IBM SPSS version 25. The minimum acceptable value for alpha is 0.70. Below this value, the internal consistency of the scale used is considered low. Conversely, the maximum expected value is 0.90^(18)^.

Finally, qualitative data from discursive responses were analyzed according to the content analysis technique proposed by Laurence Bardin, defined as a set of systematic and objective procedures for describing the content of messages capable of generating inferences about the conditions of production and reception of communications. The analysis followed the three classic stages (pre-analysis, material exploration, and treatment of results), with in-depth reading, thematic categorization of speeches, and interpretation of the findings in light of the totality and singularity of responses^(19)^.

## RESULTS

### Serious game construction

The serious game developed was called “*Rota CME*”, in the format of a board game that simulates the physical layout of a CSSD, containing five areas of this environment: reception and cleaning; disinfection; preparation; sterilization; and storage/distribution. The game begins in the reception and cleaning (decontamination) room, symbolizing the entrance to CSSD, and ends when a player reaches the storage and distribution room, which represents the last stage of the workflow. Players aim to traverse all areas of the physical layout before their opponents do.

Before the game starts, a judge (subject monitor or professor) should explain the rules of the game, making the manual available should students wish to consult it. The game can be played individually or in teams of two to five players. There is no maximum limit to the number of players per team; this number is decided by a judge, according to the number of students present.

**Figure 1:**
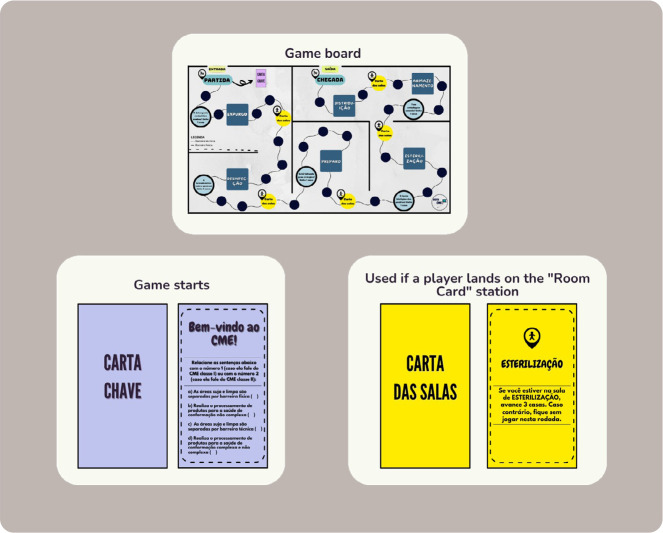
Game board and card demonstration, Recife, Pernambuco, Brazil, 2023

According to Figure 1, the game board and cards are shown. At the start of the game, all players/teams must simultaneously answer the question on the key card. They have one minute to answer and, after this time, they can consult the answer key with the judge. Players who do not correctly answer the question on the key card will not participate in the first round. The key card serves to open and start the game. The question on this card tests players’ knowledge of the types of CSSD and should only be used at the beginning of the game.

**Figure 2:**
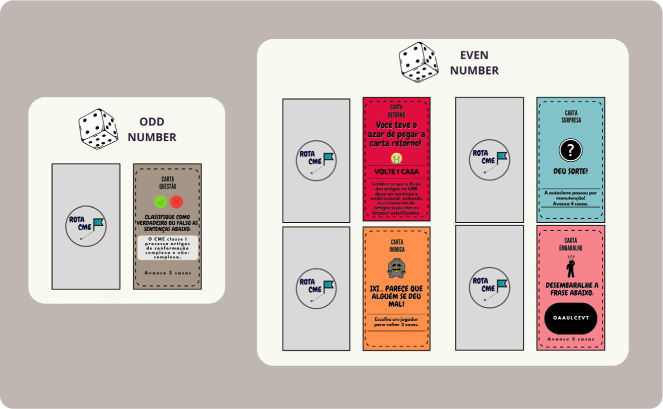
Examples of cards used in each round of the game, Recife, Pernambuco, Brazil, 2023

The game unfolds through the use of various types of cards in each round, as illustrated in Figure 2. Each round, players roll a die. If the number rolled is even, players draw a question card, which contains true or false questions. These questions cover basic concepts of CSSD, such as types of CSSDs, Spaulding classification, one-way flowcharts, regulations regarding physical plant layout according to RDC 15/2012, and the stages of processing healthcare products. Additionally, there are questions about nurses’ responsibilities in CSSD. If players answer correctly, they can advance their position.

If players’ drawn number is odd, they will pick one of the miscellaneous cards: return card, surprise card, enemy card, and shuffle card. The return card makes players go back one or more spaces; it clarifies the concept of CSSD flowchart, explaining that items should follow a continuous and unidirectional flow. With the surprise card, players can advance if it reveals a good CSSD practice; otherwise, they must go back one or more spaces. The enemy card allows players to hinder their opponents, gaining advantages over them or making them go back a few spaces. The shuffle card contains scrambled words or phrases that players must decipher within one minute; after that, they can advance spaces. All answers must be validated by a judge.

During the rounds, as players progresses along the path, they may eventually find themselves at the “Room Card” station. At this point, they must pick up a room card, which will be identified by the color yellow and separate from the others. The room card contains the name of one of the rooms on the board, and to advance the spaces, players must be lucky enough to pick the card identified by the name of the room they are currently in. Otherwise, they will not advance the spaces.

If players roll an even number and pick the question card twice in a row, in the next round, they must pick one of the miscellaneous cards, without needing to roll the die again.

### Content review by experts

The ten judges who comprised this stage of the study were female, aged between 29 and 74 years, with an average age of 46 years. Concerning their professional activity, three worked in Paraíba, two in Pernambuco, one in both Paraíba and Pernambuco, one in Ceará, one in São Paulo, and two did not provide information. As for their level of education, two were specialists, two held a master’s degree, and six held a doctoral degree. The judges’ main areas of training were surgical center and/or CSSD (n=4), hospital nursing administration (n=1), clinical practice (n=1), nursing (n=1), management (n=1), health (n=1), and statistics (n=1).

According to Table 1, judges considered the game’s content adequate (CVI ≥ 80%). The game assessment encompassed three aspects: objective; structure and presentation; and relevance. The CVI for each aspect analyzed was 100%, 100%, and 95%, respectively. Individually, 11 of the 12 items achieved a CVI of 100%, while item 3.1 (“Does the game cover the main themes about CSSD?”) obtained a CVI of 80%. The overall CVI was 98.3%, confirming the game’s validity by experts.

**Table 1:** Statistics on judges’ assessment of the “*Rota CME*” game (N=10), Recife, Pernambuco, Brazil, 2023

Assessment dimensions	CVI (%)
1 Objective	
1.1 - Does it meet academics’ learning needs?	100.0
1.2 - Is the language appropriate for the target audience?	100.0
1.3 - Does it reinforce the learning acquired in the classroom?	100.0
1.4 - Does it promote reflection on the importance of CSSD in healthcare settings?	100.0
2 Structure and presentation	
2.1 - Is this educational game appropriate for nursing students who have already experienced both the theoretical and practical aspects of the content?	100.0
2.2 - Are the game rules easy to understand?	100.0
2.3 - Is the game design intuitive and does it easily reflect the CSSD’s physical structure?	100.0
2.4 - Is the game visually appealing?	100.0
3 Relevance	
3.1 - Does the game cover the main themes about CSSD?	80.0
3.2 - Does the game encourage players to recall concepts learned in the classroom?	100.0
3.3 - Does the game clarify basic CSSD concepts?	100.0
3.4 - Does the game allow players to reflect on the importance of the work process developed by nurses in CSSD?	100.0
**Total**	98.3

*CSSD - Central Sterile Supply Department; CVI - Content Validity Index.*

In the section of the form designated for suggestions and improvements to the game, 50% of participants contributed with some feedback. The most significant suggestions concerned changes to the game’s cards and its assessment method. There were also suggestions related to modifications to the game board and its format. The comments are described below:

*I found the proposal very interesting and relevant. I suggest that return-to-home letters or equivalent should include some relevant information; for instance, “the material was not washed properly, return one space”. Replace the word “purging” with “reception and cleaning sector”, which is what is in RDC 15/2012.* (P3)

*Since I am not familiar with all the questions contained in the letters, I suggest that, in addition to basic concepts, they include questions that highlight the importance of CSSD in the proper functioning of the institution, as well as its interface with the HICC.* (P7)

*Perhaps simulating a match? That might be more enlightening.* (P4)

*There are items that we can barely see. There is a possibility of enlarging the board.* (P5)

*Congratulations on the initiative. A future suggestion is to expand the game and make it available online for other institutions.* (P10)

### Technology application with the target audience

The serious game was applied to 38 students, 32 (84.2%) of whom were female and six (15.8%) were male, aged between 19 and 26 years, with an average age of 20 years. The questionnaire aimed to assess three domains of this technology: game structure and presentation; relevance in the teaching-learning process; and work process in CSSD during the game experience.

Table 2 demonstrates psychometric variations among the analyzed domains. The “relevance in the teaching-learning process” (α=0.889) and “work process in CSSD” (α=0.882) domains show significant consistency (α≥0.70). On the other hand, the “game structure and presentation” domain showed low reliability (α=0.526), not reaching the consistency threshold, and was re-assessed and adjusted according to the suggested changes.

**Table 2:** “Game structure and presentation” domain academic assessment (N=38), Recife, Pernambuco, Brazil,

Assessment dimensions	Cronbach’s alpha
1 Structure and presentation	
1.1 - Does the game’s design easily resemble the physical layout of CSSD?	0.526
1.2 - Is the game visually appealing?	
1.3 - Are the game rules easy to understand?	
1.4 - Does the variety of cards in the game pique students’ interest in playing it?	
2 Relevance in the teaching-learning process	
2.1 - Did the game address important CSSD themes?	0.889
2.2 - Were you encouraged to put the concepts discussed earlier in class into practice?	
2.3 - Did the game help you clarify any doubts?	
2.4 - Were you able to observe CSSD’s approach to basic concepts during the game?	
3 Relevance in CSSD workflow	
3.1 - Did the game simulate situations experienced by nurses in CSSD environment?	0.882
3.2 - Did the game demonstrate how essential nurses’ work is to achieving safe and effective processing of healthcare products?	
3.3 - Does the game address nursing skills such as people management, technical and care activities, and resource control?	
3.4 - Did the game allow players to reflect on the importance of the work process developed by nurses in CSSD?	

The qualitative data related to suggestions and improvements pertain to changes in card content and changes in the game board appearance, as described in the following statements:

*Change in board design.* (P12)

*I wish the words for “untangle” were a bit more complex. Otherwise, it’s a pretty fun game.* (P21)

## DISCUSSION

This research constructed and validated a serious game aimed at teaching about the CSSD physical structure, flowchart, and functionality. The assessments conducted during the validity evidence phase, based on content and response processes, provided a critical understanding of the importance of the work process in this sector. The judges’ and target audience’s contribution allowed for a closer connection between practical reality and the theoretical conceptions of each group, broadening the understanding of the activities developed in CSSD^(20)^. This integration aimed to improve teaching and promote the development of good professional practices by linking everyday experiences with scientific evidence^(21)^.

CSSD activities are complex and essential for indirect patient care, being carried out through a standardized process in which the nursing team plays a central role in quality control and patient safety^(3,4)^. However, a descriptive-reflective study indicates that the emphasis on generalist training in nursing courses in Brazil has reduced the time dedicated to teaching the perioperative area, compromising the development of specific skills for the operating room, anesthetic recovery, and CSSD^(14)^. This reinforces the need for greater focus in this field, aligning with this study’s proposal to develop serious games for teaching CSSD^(22)^.

In the training of nurses to work in CSSD, the importance of understanding the minimum competencies required in perioperative care stands out, along with the need for educational strategies that meet the demands of new generations and address the shortage of qualified professionals in the market^(23)^.

The adoption of active learning methodologies in higher education, especially in the health field, has become established as a strategy to promote more dynamic and effective learning, replacing or complementing traditional methods. Among these resources, games stand out for encouraging active student participation, strengthening interaction, and increasing engagement with the content^(24)^.

Educational technology validity is fundamental to guaranteeing the legitimacy of the developed product. To this end, it is essential that all items are representative and clear, ensuring their proper use according to the purpose for which they were conceived. The contributions integrated into the process enable the product’s scientific construction, giving it support and effectiveness in the educational field^(25)^.

In the “*Rota CME*” game validity, all three sections, each composed of four questions, obtained a CVI higher than 80%, a value that represents consensus among experts regarding the topic or aspect assessed. This result demonstrates the positive assessment of the educational technology by experts, indicating an adequate level of agreement among opinions.

The “Does it meet academics’ learning needs?” item obtained 100% agreement among experts, a result favored by the construction of a serious game based on official guidelines and scientific literature. The ANVISA regulation, used as a theoretical reference, establishes the requirements for good practices in the processing of health products, ensuring the safety of patients and professionals involved^(13)^.

Another point positively assessed by judges was the game’s design, which was considered intuitive and alluding to CSSD’s physical structure. The representation of CSSD’s physical structure as a learning resource was highlighted by judges in the “Is the game design intuitive and does it easily reflect the CSSD’s physical structure?” item. It is important to emphasize that the construction and use of games for educational purposes must consider aspects such as didactics, playfulness, and design. The addition of protocols and theoretical concepts during teaching should not negate the attractive and fun nature of the game, which should be based on goals that combine fun and learning^(4,6)^.

CSSD organization must comply with the provisions of RDC 15/2012, requiring a physical layout divided into pre-established areas or rooms. Compliance with this requirement ensures worker safety and efficiency in their work process^(4)^. This is an important aspect, considering that one of the main purposes of the game is to clarify CSSD’s functionality for students, making them understand the infrastructure of this environment.

The cards also play an important role in expanding the theoretical content covered in the application of the activity. After all, they address topics recommended in the best practice guidelines for processing the cleaning, disinfection, and sterilization stages of healthcare products, monitoring and traceability, biosafety, and occupational risks in a competitive and playful manner^(14)^. In addition to expert assessment of the educational technology content, it is also important for the target audience to verify the need for adjustments, in order to support the development of practical and accessible material.

The first section of the form assessed the game’s suitability for the learning objectives. Studies highlight that games promote active participation, social interaction, positive emotional responses, and immediate feedback, thus favoring behavioral learning^(24,26)^. The application of games in education requires an analysis of their necessity and benefits^(27)^. In this context, the game proved to be an effective educational technology, with appropriate language, reinforcing prior knowledge and promoting understanding of the actions performed in CSSD.

Although the game was positively assessed in all three aspects analyzed, the lowest agreement rates referred to the simulation of real-life CSSD situations and reflection on nurses’ role in this context. This result may be related to the limited time for simulations, indicating the need to expand the number of situations and the interaction time in the game, bringing it closer to professional practice in CSSD^(26-29)^.

A study on validity of an educational game in biosafety at CSSD also reinforced that such tools should reflect the theme’s reality with clear language and an attractive organization, aiming for meaningful learning^(30)^. For undergraduate students, this learning is facilitated when educational interventions, such as problem simulations and real-life experiences, are based on situations from professional practice, because, in addition to promoting interactive learning, they encourage students’ proactivity and autonomy^(29)^.

Reflecting on professional practice in CSSD contributes to promoting safe practices, identifying problems, and seeking solutions in complex healthcare settings such as CSSD^(30)^. It is important to highlight that characteristics such as competitiveness and fun should be preserved, while the experience of playing the game brings lessons applicable to real-life situations.

The comments from judges and academics were carefully considered. Suggestions included adjustments to the gameplay, such as adding educational phrases to the penalty cards, and replacing terms like “purge” with “reception and cleaning”. It was also recommended to include the interrelationship between CSSD and the Hospital Infection Control Committee, highlighting their relevance in controlling healthcare-associated infections.

### Study limitations

The game’s exclusive development of a physical version stands out, preventing its practical simulation by judges and limiting interaction with the cards and understanding of the rules.

### Contributions to nursing

The “*Rota CME*” serious game proves relevant to nursing, with potential application in different higher education institutions, as it constitutes a viable didactic resource to fill gaps in nursing education. For the nursing field, the tool promotes the qualification of teaching about the CSSD structure, flow, and functionality, a sector of nurses’ work that has a direct impact on patient safety. Furthermore, the future online version could expand its reach and accessibility, integrating with students’ everyday devices, such as cell phones and computers.

## CONCLUSIONS

The “*Rota CME*” serious game had its content validated by experts and was satisfactorily assessed by nursing students. Information quality, regarding learning objectives, structure, and relevance, was ensured. The initial application demonstrated its potential to enhance teaching about CSSD, facilitating the assimilation of concepts essential to nurses’ role in the perioperative period.

Presented as a board game, the game organizes content about the physical structure, flow, and processing of healthcare products, serving as a basis for understanding practices in CSSD. It is therefore a viable teaching resource in undergraduate nursing programs, with the potential to strengthen training focused on quality and safety of care.

## Data Availability

The research data are available within the article.
