# Supplementary material for: Development and validity of a serious game for teaching Central Sterile Supply Department procedures
Source: Rev Bras Enferm. 2026 Jul 24;79:e20250453. doi: 10.1590/0034-7167-2025-0453 (PMC13403637; doi:10.1590/0034-7167-2025-0453)
Supplement: Translation

# Translation: Desenvolvimento e validação de *serious game* para ensino de Centro de Material e Esterilização

**DOI:** 10.1590/0034-7167-2025-0453pt

**Authors:** Fernanda de Andrade Ribeiro Ferreira, Felipe Almeida Sales, Yure Rodrigues Silva, Maria das Neves Figueiroa, Camilla Ribeiro Lima de Farias, Felicialle Pereira da Silva, Natália Ramos Pessoa Costa, Emanuela Batista Ferreira e Pereira

**Affiliations:** I Universidade de Pernambuco. Recife, Pernambuco, Brasil

**Keywords:** Esterilização, Avaliação de Processos em Cuidados de Saúde, Materiais de Ensino, Educação em Enfermagem, Estudo de Validação.

## Abstract

**Objetivos:**

construir e validar *serious* game para apoio ao ensino da estrutura física, fluxo e funcionalidade do Centro de Material e Esterilização no curso de graduação em enfermagem.

**Métodos:**

estudo metodológico, realizado entre abril e agosto de 2023, com a participação de dez juízes especialistas e 38 estudantes de enfermagem. A validação se baseou na estrutura do *serious game* como referencial teórico-metodológico, utilizando o Índice de Validade de Conteúdo.

**Resultados:**

o jogo “Rota CME” simula a planta física do setor para o aprendizado do aluno, e obteve Índice de Validação de Conteúdo global de 98,3%. O domínio “estrutura e apresentação do jogo” apresentou confiabilidade inferior (α=0,526), sendo ajustado conforme sugestões dos participantes.

**Conclusões:**

o jogo demonstrou evidências de validade de conteúdo e aparência, mostrando-se uma ferramenta pedagógica viável para o ensino de enfermagem, com potencial de qualificar o aprendizado na área da central de esterilização.

## INTRODUÇÃO

A atuação do enfermeiro no contexto perioperatório abrange atividades essenciais, como a organização do ambiente, o atendimento às necessidades da equipe cirúrgica e o correto processamento de produtos para a saúde. Essas ações visam proporcionar ao paciente cirúrgico assistência individualizada, segura e baseada em protocolos validados, contemplando todas as etapas da experiência assistencial^(1)^. Nesse cenário, destaca-se o papel estratégico do Centro de Material e Esterilização (CME), unidade que, embora de assistência indireta, exerce impacto direto na segurança e qualidade do cuidado prestado.

O CME tem como principal finalidade prover os diversos setores assistenciais e de diagnóstico com produtos para a saúde devidamente processados. Para tanto, exige-se o cumprimento rigoroso de boas práticas, que envolvem as etapas de pré-limpeza, limpeza, inspeção, preparo, desinfecção, esterilização, armazenamento e distribuição^(2)^. Tais processos são regulados pela Resolução de Diretoria Colegiada (RDC) nº 15/2012, que estabelece critérios baseados em evidências científicas, fundamentais para assegurar a eficácia e segurança das práticas adotadas^(3)^.

A compreensão plena da estrutura, fluxo e rotinas do CME é indispensável aos profissionais que nele atuam, sendo também imprescindível sua capacidade de identificar inconformidades que possam comprometer os resultados esperados. A infraestrutura adequada e o ordenamento físico-funcional do CME são elementos-chave para o desempenho seguro e eficiente das atividades. A organização de ambientes e o fluxo unidirecional de materiais, do sujo para o limpo, contribuem, de maneira decisiva, para prevenção de eventos adversos, como as infecções relacionadas à assistência à saúde^(4)^.

Devido à criticidade das funções exercidas nesse setor, é fundamental que a formação dos profissionais, especialmente dos enfermeiros, inclua estratégias educacionais que promovam a compreensão prática e teórica das especificidades do CME. Entretanto, a abordagem desse conteúdo no ensino de enfermagem ainda apresenta limitações. Estudo identificou que os estudantes consideram necessário o uso de metodologias mais atrativas e interativas, como vídeos educativos, para o ensino das boas práticas no CME, sobretudo devido à limitação das vivências práticas no setor, marcadas por cargas horárias reduzidas e poucas oportunidades reais de imersão^(5)^.

Tais limitações apontam lacunas na formação que podem comprometer a atuação futura do profissional. Diante desse cenário, o uso de metodologias ativas, como os *serious games*, jogos com intencionalidade educativa, tem se mostrado promissor no ensino em saúde. Ao simular o ambiente e as atividades profissionais, esses jogos favorecem a imersão dos estudantes em contextos próximos à realidade, promovendo a autonomia, o raciocínio crítico e a consolidação do conhecimento^(6,7)^.

Essa abordagem dialógica e participativa está em consonância com a Teoria da Educação Crítica e Emancipatória de Paulo Freire, que entende a educação como um processo de transformação, baseado na reflexão crítica e na ação consciente sobre a realidade vivida^(8)^. A inserção de recursos didáticos inovadores, como os jogos educativos, tem se mostrado eficaz na promoção da aprendizagem significativa, especialmente no ensino de conceitos técnicos e abstratos. Esses recursos oferecem, além de conteúdo, indicadores observáveis do desempenho discente, permitindo avaliação qualitativa e quantitativa do processo de ensino-aprendizagem^(9)^.

No entanto, a efetividade desses materiais depende de processo rigoroso de construção e validação, conduzido por especialistas da área, o que tem sido tema recorrente em pesquisas na interface entre educação e saúde^(10)^.

## OBJETIVOS

Desenvolver e validar *serious game* sobre estrutura, fluxo e funcionalidade do CME voltado a acadêmicos de enfermagem.

## MÉTODOS

### Aspectos éticos

O estudo foi conduzido de acordo com as diretrizes de ética nacionais e internacionais, e aprovado pelo Comitê de Ética em Pesquisa da Universidade de Pernambuco, cujo parecer está anexado à presente submissão. Está de acordo com o Ofício Circular nº 2/2021, que estabelece as orientações para procedimentos em pesquisas em ambiente virtual. Adicionalmente, o Termo de Consentimento Livre e Esclarecido foi obtido de todos os indivíduos envolvidos no estudo por meio de *link* para acessar o formulário de participação, o qual continha a apresentação do termo em sua primeira página de maneira que as outras partes do formulário só poderiam ser acessadas após o consentimento dos participantes. O instrumento também permitiu sugestões de alteração, e os juízes foram identificados por códigos (P1, P2...) para garantir o anonimato.

### Desenho, período e local do estudo

Trata-se de estudo metodológico, que buscou construir e validar *serious game* direcionado a estudantes do curso de enfermagem para apoiar o ensino no CME. O estudo foi realizado entre abril e agosto do ano de 2023.

Para guiar o percurso, utilizou-se o instrumento *COnsolidated criteria for REporting Qualitative research* ^(10)^. As etapas para o processo de construção e validação seguiram os passos descritos pela *American Educational Research Association*, a qual considera as seguintes evidências de validade: evidência baseada no conteúdo do teste; evidência baseada em processos de resposta; evidência baseada na estrutura interna; e evidência baseada em relações com outras variáveis^(11)^. Dessa forma, foram verificadas as evidências com base no conteúdo da tecnologia educacional e nos processos de resposta. A pesquisa em si foi desenvolvida em três etapas: construção do *serious game*; avaliação do conteúdo junto a especialistas; e aplicação da tecnologia com o público-alvo.

Como base teórica, o referencial do *serious game* foi a Teoria da Educação Crítica e Emancipatória de Paulo Freire^(8)^, e os conteúdos presentes no jogo foram definidos de acordo com as diretrizes estabelecidas pelos seguintes documentos técnicos: RDC nº 15/2012 da Agência Nacional de Vigilância Sanitária do Brasil (ANVISA)^(12)^; Diretrizes de Práticas em Enfermagem Cirúrgica e Processamento de Produtos para a Saúde da Associação Brasileira de Enfermeiros de Centro Cirúrgico, Recuperação Anestésica e Centro de Material e Esterilização^(13)^; e a norma ABNT NBR ISO 17665-2^(14)^. Tais documentos estabelecem os requisitos de boas práticas para o processamento de produtos para a saúde, e auxiliaram na condução dos seguintes temas abordados no *serious game:* estrutura física, funcionalidade e fluxograma do CME.

Já o referencial metodológico para criação do recurso didático na forma de jogo foi baseado na estrutura do *serious game*. Este tipo de jogo tem como principal objetivo ensinar, utilizando-se diversos recursos e materiais multimídia^(7)^.

### População e amostra; critérios de inclusão e exclusão

Participaram desta etapa dez juízes, respeitando a recomendação de Pasquali de, pelo menos, seis especialistas na temática para avaliação do conteúdo proposto^(15)^. Os juízes atenderam aos seguintes critérios: categoria profissional; produção científica ligada à área do CME; e nível de especialização. Inicialmente, realizou-se a busca sistematizada na literatura científica para identificar autores com publicações relevantes sobre a temática do CME.

Com base nessa análise, foram selecionados especialistas, os quais foram convidados a participar do estudo por meio de contato eletrônico, incluindo informações detalhadas sobre os objetivos da pesquisa e os critérios de participação. Além disso, os juízes foram selecionados por meio do tipo de amostragem não probabilística por bola de neve, de maneira que, após a avaliação, solicitou-se ao primeiro juiz que indicasse novos participantes, que realizaram novas indicações até que a amostra fosse alcançada.

Para certificar que os juízes indicados contemplavam os critérios adotados nesta pesquisa, foi verificado o currículo disponibilizado na Plataforma *Lattes* do Conselho Nacional de Desenvolvimento Científico e Tecnológico.

O público-alvo para aplicação da tecnologia foram estudantes que cursavam o quinto período do curso de enfermagem em uma universidade pública do nordeste brasileiro.

### Protocolo do estudo

Na segunda etapa do estudo, o conteúdo da tecnologia educacional foi avaliado por juízes-especialistas quanto à clareza e pertinência. Cada juiz recebeu, via e-mail, uma carta convite para participação na pesquisa com a descrição do objetivo do estudo, forma de condução, informações acerca da origem do material a ser avaliado, e arquivo em PDF contendo imagens das cartas e do tabuleiro em alta definição junto com o formulário. O formulário foi composto de quatro seções: (1) caracterização da amostra; (2) objetivo de aprendizagem do jogo; (3) estrutura e apresentação do jogo; e (4) relevância no processo de ensino-aprendizagem. A avaliação foi realizada a partir de perguntas com cinco opções de resposta, graduadas em uma escala do tipo Likert, que continham as seguintes alternativas: concordo; concordo parcialmente; não concordo nem discordo; discordo parcialmente; e discordo.

Na terceira etapa, buscou-se verificar as evidências de validade baseadas em processos de resposta. Para tanto, foi realizada a aplicação da tecnologia entre o público-alvo. A aplicação do jogo ocorreu no laboratório de habilidades da instituição, no decorrer de quatro dias, de maneira que os estudantes foram divididos em grupos compostos por dez pessoas, a fim de garantir maior aproveitamento.

O roteiro da intervenção educativa ocorreu de forma semelhante em cada um dos dias. Inicialmente, houve uma abordagem teórico-prática em que as áreas do CME foram apresentadas por meio de estações que representaram o expurgo, locais de preparo, esterilização e guarda de insumos. Em seguida, o jogo foi aplicado entre os estudantes por um docente responsável pela disciplina e por uma acadêmica de enfermagem, as quais estiveram envolvidas na produção da tecnologia educacional. Por fim, os acadêmicos avaliaram o *serious game* quanto à sua estrutura e apresentação, relevância no processo de ensino-aprendizagem, e pertinência no processo de trabalho do CME. Para isso, os dados foram coletados em ambiente virtual com o uso de questionário do *Google Forms^®^*.

A coleta de dados foi feita por meio de instrumento de opinião em escala do tipo Likert com perguntas objetivas que continham cinco opções de resposta: concordo; concordo parcialmente; não concordo nem discordo; discordo parcialmente; e discordo. O instrumento também disponibilizou espaço para que os graduandos pudessem propor alterações, caso julgassem necessário.

### Análise dos resultados e estatística

Os resultados foram analisados em uma planilha do *Microsoft Excel^®^* para o cálculo dos Índices de Validade de Conteúdo de Itens (IVC-I) e em Escala (IVC-S) global^(16)^. O IVC-I foi obtido pela proporção de juízes que responderam “concordo” ou “concordo parcialmente” em cada item. Por sua vez, o IVC-S foi calculado pela média das proporções de itens considerados válidos por cada juiz. Adotou-se um índice de concordância mínimo de 80% para validação^(17)^.

Para avaliar o questionário aplicado aos estudantes, foi obtido o coeficiente de consistência interna alfa de Cronbach por bloco como critério de avaliação sobre a pertinência do item. Os dados foram digitados em uma planilha, e o programa utilizado para obtenção dos cálculos estatísticos foi o IBM SPSS na versão 25. O valor mínimo aceitável para o alfa é 0,70. Abaixo desse valor, a consistência interna da escala utilizada é considerada baixa. Em contrapartida, o valor máximo esperado é 0,90^(18)^.

Por fim, os dados qualitativos das respostas discursivas foram analisados conforme a técnica de análise de conteúdo proposta por Laurence Bardin, definida como um conjunto de procedimentos sistemáticos e objetivos de descrição do conteúdo das mensagens capazes de gerar inferências sobre as condições de produção e recepção das comunicações. A análise seguiu as três etapas clássicas (pré-análise, exploração do material e tratamento dos resultados), com leitura aprofundada, categorização temática das falas, e interpretação dos achados à luz da totalidade e singularidade das respostas^(19)^.

## RESULTADOS

### Construção do *serious game*

O *serious game* desenvolvido foi denominado “Rota CME”, no formato de um jogo de tabuleiro que simula a planta física do CME, contendo cinco áreas deste ambiente: recepção e limpeza; desinfecção; preparo; esterilização; e armazenamento/distribuição. O jogo tem início na sala de recepção e limpeza (expurgo), simbolizando a entrada no CME, e é finalizado quando o jogador chega à sala de armazenamento e distribuição, a qual representa a última etapa do fluxograma do processo de trabalho. O objetivo do jogador é percorrer por todos os ambientes da planta física antes que os seus oponentes o façam.

Antes do início da partida, o juiz (monitor da disciplina ou professor) deve explicar as regras do jogo disponibilizando o manual, caso os alunos queiram consultar. O jogo pode ser vivenciado individualmente ou em times, com um número de dois a cinco jogadores ou times. Não há limite máximo para a quantidade de jogadores por time; essa quantidade é decidida pelo juiz, de acordo com o número de alunos presentes.

De acordo com a Figura 1 estão demonstrados o tabuleiro e cartas do jogo. Ao iniciar o jogo, todos os jogadores/times devem responder, simultaneamente, à questão da carta-chave. Eles têm o intervalo de um minuto para responder e, após este tempo, podem consultar o gabarito com o juiz. O jogador que não responder corretamente à pergunta da carta-chave não participará da primeira rodada. A carta-chave tem a função de abrir e iniciar o jogo. A pergunta contida nesta carta testa o conhecimento do jogador acerca dos tipos de CME, devendo ser usada apenas no início do jogo.

**Figura 1:** Demonstração do tabuleiro e cartas do jogo, Recife, Pernambuco, Brasil, 2023

O jogo se desenvolve com a utilização de variados tipos de cartas a cada rodada do jogo, conforme exemplificado na Figura 2. A cada rodada, o jogador sorteará o dado. Se o número sorteado for par, o jogador pegará a carta questão, que contém perguntas com respostas de verdadeiro ou falso. As perguntas desse tipo de carta abordam conceitos básicos do CME, como tipos de CME, classificação de Spaulding, fluxograma unidirecional, normas sobre a planta física segundo a RDC nº 15/2012 e as etapas do processamento dos produtos de saúde. Além disso, possui perguntas sobre as atribuições do enfermeiro no CME. Se o jogador responder corretamente, ele poderá avançar nas casas.

**Figura 2:** Exemplos de cartas usadas a cada rodada do jogo, Recife, Pernambuco, Brasil, 2023

Se o número sorteado pelo jogador for ímpar, ele pegará uma das cartas diversas, nomeadamente carta retorno, carta surpresa, carta inimiga e carta embaralho. A carta retorno faz com que o jogador volte uma ou mais casas; ela esclarece o conceito do fluxograma do CME, explicando que os artigos devem seguir um fluxo contínuo e unidirecional. Com a carta surpresa, o jogador poderá avançar se ela trouxer uma boa prática do CME, caso contrário, ele deverá retornar uma ou mais casas. A carta inimiga permite que o jogador atrapalhe seus oponentes, tirando vantagens sobre eles ou fazendo-os voltar algumas casas. A carta embaralho contém palavras ou frases embaralhadas que devem ser desvendadas pelo jogador no intervalo de um minuto; após isso, ele poderá avançar nas casas. Todas as respostas devem ser validadas pelo juiz.

Durante as rodadas, enquanto o jogador avança no percurso, eventualmente, ele poderá se encontrar na estação “Carta das Salas”. Neste momento, ele deverá pegar uma carta das salas, que estará identificada pela cor amarela e separada das demais. A carta das salas contém o nome de um dos ambientes do tabuleiro e, para avançar nas casas, o jogador deve ter a sorte de pegar a carta identificada pelo nome do ambiente em que se encontra no momento. Caso contrário, não avançará nas casas.

Se o jogador sortear um número par e pegar a carta questão por duas vezes consecutivas, na rodada seguinte, ele deverá pegar uma das cartas diversas, sem a necessidade de sortear o dado novamente.

### Avaliação do conteúdo junto a especialistas

Os dez juízes que compuseram esta etapa do estudo eram do sexo feminino, possuíam faixa etária entre 29 e 74 anos, com média de idade de 46 anos. Em relação ao estado de atuação profissional, três atuavam na Paraíba, dois, em Pernambuco, um, na Paraíba e em Pernambuco, um, no Ceará, um, em São Paulo, e dois não informaram. Sobre o nível de formação, dois eram especialistas, dois, mestres, e seis, doutores. As áreas de maior formação dos juízes foram centro cirúrgico e/ou CME (n=4), administração de enfermagem hospitalar (n=1), clínica (n=1), enfermagem (n=1), gestão (n=1), saúde (n=1) e estatística (n=1).

De acordo com a Tabela 1, os juízes consideraram o conteúdo do jogo adequado (IVC ≥ 80%). A avaliação do jogo contemplou três aspectos: objetivo; estrutura e apresentação; e relevância. O IVC para cada aspecto analisado foi de 100%, 100% e 95%, respectivamente. Individualmente, 11 dos 12 itens alcançaram um IVC de 100%, enquanto o item 3.1 (“O jogo abrange as principais temáticas sobre o CME?”) obteve um IVC de 80%. O IVC global foi de 98,3%, confirmando a validação do jogo pelos especialistas.

**Tabela 1:** Estatística da avaliação dos juízes sobre o jogo “Rota CME” (N=10), Recife, Pernambuco, Brasil, 2023

Dimensões de avaliação	IVC (%)
1 Objetivo	
1.1 - Atende às necessidades de aprendizagem dos acadêmicos?	100.0
1.2 - A linguagem está apropriada para o público-alvo?	100.0
1.3 - Reforça o aprendizado adquirido na sala de aula?	100.0
1.4 - Promove reflexão sobre a importância do CME nos ambientes de saúde?	100.0
2 Estrutura e apresentação	
2.1 - O jogo educativo é apropriado para acadêmicos de enfermagem que tenham vivenciado o conteúdo teórico e prático?	100.0
2.2 - As regras do jogo são de fácil compreensão?	100.0
2.3 - O *design* do jogo é intuitivo e remete facilmente à estrutura física do CME?	100.0
2.4 - A aparência do jogo é atrativa?	100.0
3 Relevância	
3.1 - O jogo abrange as principais temáticas sobre o CME?	80.0
3.2 - O jogo estimula o jogador a lembrar dos conceitos aprendidos em sala de aula?	100.0
3.3 - O jogo esclarece conceitos básicos do CME?	100.0
3.4 - O jogo permite ao jogador fazer uma reflexão sobre a importância do processo de trabalho desenvolvido pelo enfermeiro no CME?	100.0
**Total**	98.3

*CME - Centro de Material e Esterilização; IVC - Índice de Validação de Conteúdo.*

No espaço do formulário destinado a sugestões e melhorias do jogo, 50% dos participantes contribuíram com alguma colocação. As sugestões mais significativas foram em relação às mudanças na carta e à forma de avaliação do jogo. Também houve sugestões relacionadas às modificações no tabuleiro e ao formato do jogo. As falas estão descritas abaixo:

*Achei a proposta muito interessante e relevante. Sugiro que, nas cartas de voltar casas ou equivalente, ter alguma informação relevante; por exemplo, “o material não foi lavado adequadamente, volte uma casa”. Trocar a palavra “expurgo” com “setor de recepção e limpeza”, que é o que tem na RDC n^o^ 15/2012.* (P3)

*Por não conhecer todas as perguntas contidas nas cartas, sugiro que, além de conceitos básicos, elas tragam questões que disseminem a importância da CME no bom funcionamento da instituição, bem como a interface com a CCIH.* (P7)

*Talvez simular uma partida? Poderia ser mais esclarecedor.* (P4)

*Existe item que quase não visualizamos. Há possibilidade de ampliar o tabuleiro.* (P5)

*Parabéns pela iniciativa. Uma sugestão futura é ampliar o jogo e disponibilizá-lo online para outras instituições.* (P10)

### Aplicação da tecnologia com o público-alvo

O *serious game* foi aplicado com 38 estudantes, sendo 32 (84,2%) do sexo feminino e seis (15,8%) do sexo masculino, com faixa etária entre 19 e 26 anos, e com a média de idade de 20 anos. As perguntas do formulário tinham como objetivo avaliar três domínios da tecnologia: estrutura e apresentação do jogo; relevância no processo de ensino-aprendizagem; e processo de trabalho no CME na vivência do jogo.

A Tabela 2 demonstra variações psicométrica entre os domínios analisados. Os domínios “relevância no processo de ensino-aprendizagem” (α=0,889) e “processo de trabalho no CME” (α=0,882) revelam uma consistência significativa (α≥0,70). Por outro lado, o domínio “estrutura e apresentação do jogo” apresentou baixa confiabilidade (α=0,526), não atingindo o limiar de consistência, sendo reavaliado e ajustado conforme as alterações sugeridas.

**Tabela 2:** Avaliação dos acadêmicos sobre o domínio “estrutura e apresentação do jogo” (N=38), Recife, Pernambuco, Brasil, 2023

Dimensões de avaliação	Alfa de Cronbach
1 Estrutura e apresentação	
1.1 - O *design* do jogo remete facilmente à planta física do CME?	0.526
1.2 - A aparência do jogo é atrativa?	
1.3 - As regras do jogo são de fácil compreensão?	
1.4 - A variedade de cartas do jogo desperta interesse no estudante em querer jogá-lo?	
2 Relevância no processo de ensino-aprendizagem	
2.1 - O jogo abordou temáticas importantes do CME?	0.889
2.2 - Você foi estimulado a colocar em prática os conceitos discutidos anteriormente em sala de aula?	
2.3 - O jogo permitiu que você esclarecesse dúvidas?	
2.4 - Você conseguiu observar a abordagem de conceitos básicos do CME durante o jogo?	
3 Pertinência no processo de trabalho do CME	
3.1 - O jogo simulou situações vivenciadas pelos enfermeiros no ambiente do CME?	0.882
3.2 - O jogo demonstrou como o trabalho do enfermeiro é essencial para que se obtenha um processamento de produtos para saúde seguro e eficaz?	
3.3 - O jogo aborda competências do enfermeiro como gestão de pessoas, atividades técnico-assistenciais e controle de recursos?	
3.4 - O jogo permitiu ao jogador fazer uma reflexão sobre a importância do processo de trabalho desenvolvido pelo enfermeiro no CME?	

Os dados qualitativos relacionados a sugestões e melhorias estão relacionados às mudanças no conteúdo das cartas e mudanças na aparência do tabuleiro, como descrito nas seguintes falas:

*Mudança no design do tabuleiro*. (P12)

*Gostaria que as palavras para desembaraçar fossem um pouco mais complexas. Além disso, é um jogo bastante divertido*. (P21)

## DISCUSSÃO

Esta pesquisa construiu e validou *serious game* voltado ao ensino sobre a estrutura física, o fluxograma e a funcionalidade do CME. As avaliações realizadas nas etapas de evidência de validade, com base no conteúdo e nos processos de resposta, proporcionaram uma percepção crítica acerca da importância do processo de trabalho nesse setor. A contribuição dos juízes e do público-alvo possibilitou a aproximação entre a realidade prática e as concepções teóricas de cada grupo, ampliando a compreensão sobre as atividades desenvolvidas no CME^(20)^. Essa integração teve como propósito qualificar o ensino e promover o desenvolvimento de boas práticas profissionais, ao articular experiências do cotidiano com evidências científicas^(21)^.

As atividades do CME são complexas e essenciais para o cuidado indireto ao paciente, sendo realizadas por meio de processo padronizado, em que a equipe de enfermagem desempenha papel central no controle de qualidade e na segurança do paciente^(3,4)^. Contudo, estudo descritivo-reflexivo aponta que a ênfase na formação generalista nos cursos de enfermagem no Brasil tem reduzido o tempo destinado ao ensino da área perioperatória, comprometendo o desenvolvimento de habilidades específicas para o centro cirúrgico, recuperação anestésica e CME^(14)^. Isso reforça a necessidade de maior enfoque nesse campo, alinhando-se à proposta deste estudo de desenvolver *serious game* para o ensino do CME^(22)^.

Na formação de enfermeiros para atuação no CME, destaca-se a importância da compreensão das competências mínimas exigidas no cuidado perioperatório, aliada à necessidade de estratégias educacionais que atendam às demandas das novas gerações e supram a escassez de profissionais qualificados no mercado^(23)^.

A adoção de metodologias ativas no ensino superior, especialmente na área da saúde, tem se consolidado como estratégia para promover uma aprendizagem mais dinâmica e eficaz, ao substituir ou complementar métodos tradicionais. Entre esses recursos, os jogos se destacam por estimular a participação ativa dos estudantes, fortalecer a interação e ampliar o engajamento com o conteúdo^(24)^.

A validação de tecnologia educacional é fundamental para garantir a legitimidade do produto desenvolvido. Para isso, é essencial que todos os itens sejam representativos e apresentem clareza, assegurando sua adequada utilização conforme a finalidade para a qual foram concebidos. As contribuições integradas ao processo possibilitam a construção científica do produto, conferindo-lhe respaldo e eficácia no âmbito educativo^(25)^.

Na validação do jogo “Rota CME”, todas as três seções, cada uma composta por quatro perguntas, obtiveram um IVC superior a 80%, valor que representa o consenso entre especialistas quanto ao tópico ou aspecto avaliado. Esse resultado evidencia a avaliação positiva da tecnologia educacional pelos especialistas, indicando nível adequado de concordância entre os pareceres.

O item “Atende às necessidades de aprendizagem dos acadêmicos?” obteve 100% de concordância entre os especialistas, resultado favorecido pela construção do *serious game* com base em diretrizes oficiais e literatura científica. A regulamentação da ANVISA, utilizada como referência teórica, estabelece os requisitos de boas práticas para o processamento de produtos para a saúde, assegurando a segurança do paciente e dos profissionais envolvidos^(13)^.

Outro ponto avaliado positivamente pelos juízes foi o *design* do jogo, o qual foi considerado intuitivo e alusivo à estrutura física do CME. Observa-se a representação da estrutura física do CME como um recurso de aprendizado, o que foi evidenciado pelos juízes no item “O *design* do jogo é intuitivo e remete facilmente à estrutura física do CME?”. É importante destacar que a construção e utilização de jogo para fins educacionais deve levar em consideração aspectos como didática, ludicidade e *design*. A adição de protocolos e conceitos teóricos durante o ensino não deve anular o fenômeno atrativo e divertido do jogo, o qual deve se basear em metas que unem diversão e aprendizado^(4,6)^.

A organização do CME deve estar alinhada ao que preconiza a RDC nº 15/2012, necessitando apresentar uma planta física dividida em áreas ou salas pré-estabelecidas. O cumprimento desse requisito proporciona segurança ao trabalhador e eficiência no seu processo de trabalho^(4)^. Esse é um aspecto importante, levando em consideração que uma das principais finalidades do jogo é esclarecer ao acadêmico a funcionalidade do CME, fazendo-o compreender a infraestrutura desse ambiente.

As cartas também cumprem um importante papel em ampliar o conteúdo teórico abordado na aplicação da dinâmica. Afinal, elas tratam de temas recomendados nas diretrizes de práticas para o processamento das etapas de limpeza, desinfecção e esterilização de produtos para saúde, monitoramento e rastreabilidade, biossegurança, e riscos ocupacionais de maneira competitiva e lúdica^(14)^. Além da avaliação do conteúdo da tecnologia educacional por especialistas, também é importante que o público ao qual se destina o produto verifique a necessidade de adequações, a fim de subsidiar o desenvolvimento de um material prático e acessível.

A primeira seção do formulário avaliou a adequação do jogo aos objetivos de aprendizagem. Estudos destacam que jogos promovem participação ativa, interação social, respostas emocionais positivas e *feedback* imediato, favorecendo a aprendizagem comportamental^(24,26)^. A aplicação de jogos no ensino exige análise de sua necessidade e benefícios^(27)^. Nesse contexto, o jogo demonstrou ser uma tecnologia educacional eficaz, com linguagem apropriada, reforçando conhecimentos prévios e promovendo a compreensão das ações realizadas no CME.

Embora o jogo tenha sido avaliado positivamente nos três aspectos analisados, os menores índices de concordância se referiram à simulação de situações reais do CME e à reflexão sobre o papel do enfermeiro nesse contexto. Tal resultado pode estar relacionado ao tempo limitado para simulações, indicando a necessidade de ampliar o número de situações e o tempo de interação no jogo, aproximando-o da prática profissional no CME^(26-29)^.

Estudo sobre a validação de jogo educativo em biossegurança no CME também reforçou que tais ferramentas devem refletir a realidade do tema com linguagem clara e organização atrativa, visando uma aprendizagem significativa^(30)^. Para o público universitário, essa aprendizagem é facilitada quando as intervenções educativas, como simulações de problemas e vivências reais, baseiam-se em situações da prática profissional, pois, além de promover um aprendizado interativo, estimulam a proatividade e autonomia do acadêmico^(29)^.

A reflexão sobre a prática profissional no CME contribui para promoção de ações seguras, identificação de problemas e busca pelas soluções em contextos de saúde complexos como o CME^(30)^. Destaca-se que características como competitividade e diversão devem ser preservadas, ao passo que a vivência do jogo traz aprendizados aplicados à realidade.

Os comentários dos juízes e acadêmicos foram cuidadosamente considerados. As sugestões envolveram ajustes na jogabilidade, como a inclusão de frases educativas nas cartas de penalização, e a substituição de termos, como “expurgo” por “recepção e limpeza”. Também foi recomendada a inserção da inter-relação entre o CME e a Comissão de Controle de Infecção Hospitalar, destacando sua relevância no controle das infecções relacionadas à assistência à saúde.

### Limitações do estudo

Destaca-se o desenvolvimento exclusivo da versão física do jogo, o que impediu sua simulação prática pelos juízes, limitando a interação com as cartas e a compreensão das regras.

### Contribuições para a área da enfermagem

O *serious game* “Rota CME” se mostra relevante para a área da enfermagem, com potencial de aplicação em diferentes instituições de ensino superior, uma vez que se configura como recurso didático viável para sanar lacunas na formação em enfermagem. Para a área da enfermagem, a ferramenta promove a qualificação do ensino sobre a estrutura, o fluxo e a funcionalidade do CME, setor de atuação do enfermeiro que exerce impacto direto na segurança do paciente. Além disso, a futura versão *online* poderá ampliar seu alcance e acessibilidade, integrando-se aos dispositivos cotidianos dos estudantes, como celulares e computadores.

## CONCLUSÕES

O *serious game* “Rota CME” teve seu conteúdo validado por especialistas, e foi satisfatoriamente avaliado por acadêmicos de enfermagem. A qualidade das informações, quanto aos objetivos de aprendizagem, estrutura e relevância, foi assegurada. A aplicação inicial demonstrou seu potencial para qualificar o ensino sobre o CME, facilitando a assimilação de conceitos essenciais à atuação do enfermeiro no perioperatório.

Em formato de tabuleiro, o jogo organiza conteúdos sobre estrutura física, fluxo e processamento de produtos para a saúde, servindo como base para compreensão das práticas no CME. Configura-se, assim, como recurso didático viável na graduação em enfermagem, com potencial para fortalecer a formação voltada à qualidade e segurança da assistência.

## Data Availability

Os dados de pesquisa estão disponíveis no corpo do artigo.
